# Innate, T-, and B-Cell Responses in Acute Human Zika Patients

**DOI:** 10.1093/cid/cix732

**Published:** 2017-08-17

**Authors:** Lilin Lai, Nadine Rouphael, Yongxian Xu, Muktha S Natrajan, Allison Beck, Mari Hart, Matthew Feldhammer, Amanda Feldpausch, Charles Hill, Henry Wu, Jessica K Fairley, Pamela Lankford-Turner, Nicole Kasher, Patrick Rago, Yi-Juan Hu, Srilatha Edupuganti, Shital M Patel, Kristy O Murray, Mark J Mulligan, Emory Zika Patient Study Team, Briyana Domjahn, Dongli Wang, Mary Bower, Rijalda Deovic, Sree Aramgam, Sara Jo Johnson, Dean Kleinhenz, JoAnn Sadowski, Talib Sirajud-Deen, Jesse Waggoner

**Affiliations:** 1 Hope Clinic of the Emory Vaccine Center, Division of Infectious Diseases, Department of Medicine, School of Medicine, Emory University, Decatur; 2 Department of Pathology, School of Medicine, Emory University, Atlanta, Georgia; 3 Georgia Department of Public Health, Emory University, Atlanta, Georgia; 4 Emory TravelWell Center, Division of Infectious Diseases, Department of Medicine, School of Medicine, Emory University, Atlanta, Georgia; 5 Department of Biostatistics and Bioinformatics, Rollins School of Public Health, Emory University, Atlanta, Georgia; 6 Section of Infectious Diseases, Departments of Medicine and Molecular Virology and Microbiology; 7 Pediatrics-Tropical Medicine, Texas Children’s Hospital, Baylor College of Medicine, Houston; 8 Hope Clinic of the Emory Vaccine Center, Division of Infectious Diseases, Department of Medicine, School of Medicine, Emory University, Decatur, Georgia; 9 Hope Clinic of the Emory Vaccine Center, Division of Infectious Diseases, Department of Medicine, School of Medicine, Emory University, Decatur, Georgia and Emory TravelWell Center, Division of Infectious Diseases, Department of Medicine, School of Medicine, Emory University, Atlanta, Georgia

**Keywords:** Zika, immunity, pregnancy, viral persistence, flavivirus

## Abstract

**Background:**

There is an urgent need for studies of viral persistence and immunity during human Zika infections to inform planning and conduct of vaccine clinical trials.

**Methods:**

In 5 returned US travelers with acute symptomatic Zika infection, clinical features, viral RNA levels, and immune responses were characterized.

**Results:**

Two pregnant, flavivirus-experienced patients had viral RNA persist in plasma for >44 and >26 days. Three days after symptom onset, transient increases in proinflammatory monocytes began followed at 5 days by transient decreases in myeloid dendritic cells. Anti-Zika virus immunoglobulin M was detected at day 7 after symptom onset, persisted beyond 103 days, and remained equivocal through day 172. Zika virus–specific plasmablasts and neutralizing antibodies developed quickly; dengue virus–specific plasmablasts and neutralizing antibodies at high titers developed only in flavivirus-experienced patients. Zika virus– and dengue virus–specific memory B cells developed in both flavivirus-naive and -experienced patients. CD4+ T cells were moderately activated and produced antiviral cytokines after stimulation with Zika virus C, prM, E, and NS5 peptides in 4/4 patients. In contrast, CD8+ T cells were massively activated, but virus-specific cells that produced cytokines were present in only 2/4 patients assessed.

**Conclusions:**

Acute infections with Zika virus modulated antigen-presenting cell populations early. Flavivirus-experienced patients quickly recalled cross-reactive MBCs to secrete antibodies. Dengue virus–naive patients made little dengue-specific antibody but developed MBCs that cross-reacted against dengue virus. Zika virus–specific functional CD4+ T cells were readily detected, but few CD8+ T cells specific for the tested peptides were found.

## Teaser

Understanding the immune response during acute Zika in humans will aid vaccine design and testing. In 5 acute patients, including 2 pregnant women, viral levels and innate, T-, and B-cell responses against Zika or dengue viruses are described.

Each year hundreds of thousands of US travelers return from countries where the Zika virus (ZIKV) epidemic has occurred. As of 19 July 2017, 5120 US travelers had been diagnosed with Zika [1]. ZIKV infections in pregnant women may produce the congenital Zika syndrome in fetuses [2–4]. ZIKV infections in adults are linked to rare, serious neurologic complications including Guillain-Barré syndrome [5–7]. Cardiac complications were recently reported [8, 9].

Studies of human ZIKV infections (including flavivirus-experienced or –naive hosts and pregnant women) are needed to optimally inform testing of Zika vaccines [10–14]. Immune responses to ZIKV infection were evaluated in a macaque model [15], but there are remarkably few published human studies of fundamental parameters such as the magnitude, kinetics, quality, and specificity of innate, effector, and memory cellular responses to ZIKV [16–18]. Dengue virus (DENV) immunity may be a useful point of reference; however, ZIKV has only a single serotype [16], which is an advantage for ZIKV vaccine development and testing. Human studies of DENV have identified early host responses such as loss of myeloid dendritic cells (DC) and increases in CD14+CD16+ monocytes, followed by robust expansions of plasmablasts [19]. After yellow fever (YF)-17D immunization, an increase in CD38+HLA-DR+ activated CD8+ T cells was detected in all vaccine recipients and was associated with transient plasma viral RNA detection. The peak of the CD8+ T-cell response was observed at day 15 after immunization, when 4%–13% of CD8+ T cells coexpressed CD38 and HLA-DR[20].

Here, we present immune response and viral persistence data from 5 acute ZIKV infections.

## METHODS

Patients with confirmed ZIKV infections were identified and offered participation in an Emory University Institutional Review Board–approved protocol to study emerging infections. Time points in this convenience sample were designated as the days post onset of symptoms (DPO).

Quantitative real-time polymerase chain reaction (qRT-PCR) for Zika diagnosis or ZIKV persistence was performed on multiple body fluids as described [21]. For patient D, sera rather than plasma were assayed [22]; for simplicity the word plasma is used.

The contemporary ZIKV strain used for focus reduction neutralization test (FRNT) and Zika IgM antibody capture enzyme-linked immunosorbent assay (Zika MAC-ELISA) (PRVABC59 [KU501215.1]) was provided by the US Centers for Disease Control and Prevention. DENV-1 (Hawaii), DENV-2 (New Guinea C), and DENV-4 (H241) viruses were provided by BEI Resources (https://www.beiresources.org). DENV-3 (Sleman/78) was provided by Jens Wrammert (Emory University). ZIKV was passaged by infecting Vero cells (American Type Culture Collection; CRL-1586) at a multiplicity of infection of 0.05 in serum-free minimum essential medium (MEM; Life Technologies Gibco). After a 1-hour infection at 37°C, MEM supplemented with 10% (vol/vol) fetal bovine serum (FBS) and 1% antibiotic/antimycotic (Corning MT30004CI) were added. Upon observation of severe cytopathic effect at day 3, supernatants were collected and spun down at 930×*g* for 10 minutes at 4°C. Virus-containing supernatant was supplemented with an additional 10% (vol/vol) FBS before freezing at –80°C. DENV-1–4 viruses were also passaged as described above, and virus-containing supernatants were collected at 8–11 days post-infection. Titers of the passaged viruses were determined by focus forming assay [23]. For the IgM antibody-capture enzyme-linked immunosorbent assay (MAC-ELISA) immunoglobulin (Ig) M assay [24], viral stocks were inactivated with 0.1% beta-propiolactone overnight. Antigen was produced by spinning inactivated supernatants in Amicon Ultra-15 centrifugal filters at 3500×*g* for 25 minutes at 4°C.

ZIKV lysate for B cell enzyme-linked immunospot (ELISpot) assays was prepared from Vero cells infected as above. After cell-free virus was harvested, the remaining adherent cells and cell pellet from the supernatant were washed twice with phosphate-buffered solution and resuspended in radioimmunoprecipitation assay (RIPA) buffer (Abcam; Ab156034) supplemented with protease inhibitor (Thermo Fisher Scientific; 87785) and phosphatase inhibitor (Biovision; K275-1). Mock lysate was prepared similarly from uninfected cells. Protein concentrations were quantified using NanoDrop 8000 (ThermoFisher). DENV-1–4 recombinant E proteins were purchased from CTK Diagnostics (A2301, DENV-1 VN/BID-V949/2007; A2302, DENV-2 GWL39 IND-01; A2303, DENV-3 US/BID-V1090/1998; and A2304, DENV-4 341750).

Peptide pools for intracellular cytokine staining (ICS) assays consisted of 15-mers with 11 amino acid overlaps spanning ZIKV proteins C (28 peptides), prM (40 peptides), and E (pool 1, 62 peptides; pool 2, 62 peptides) (JPT Peptide Technologies, Berlin, Germany; GenBank: KU527068). Pools of 10 overlapping 15-mer peptides derived from ZIKV NS5 (GenBank: KU321639.1) were provided by D. H. O’Connor (University of Wisconsin–Madison) and synthesized using GenScript (Piscataway, New Jersey) [15].

Further experimental methods for immune phenotyping of innate cells by flow cytometry, focus reduction neutralization test (FRNT), MAC-ELISA, West Nile virus (WNV) ELISA, B-cell ELISpot, and intracellular cytokine staining (ICS) are provided in the Supplementary Materials.

## RESULTS

Five returned travelers with acute Zika confirmed by qRT-PCR were enrolled between DPOs 3 and 11 and followed for up to 11 months (Table 1). Clinical signs and symptoms included rash (5/5), myalgia (4/5), fever (4/5), prolonged fatigue (4/5), joint pain (2/5), and conjunctivitis (1/5); most resolved in 1–3 weeks (Table 2).

**Table 1.** Demographics, Characteristics, and Antibody Responses for 5 Acute Zika Patients: Categorization as Flavivirus Experienced or Flavivirus Naive

Patient (age, gender, country visited, reason)	Reported Previous Flavivirus Exposure; DPO when ZIKV PCR Diagnosis Made	Flavivirus Exposure Category (Pregnancy Status)	DPO	IgM (MAC- ELISA)^a^	Neutralizing Ab to ZIKV and DENVs (FRNT_50_ titer)					IgG (ELISA titer)^b^
				ZIKV	ZIKV	DENV-1	DENV-2	DENV-3	DENV-4	WNV
**A-23 (27, male, Belize, vacation**)	None; 3	Naive (NA)	5	0.7	<30	<30	<30	<30	<30	<10
			9	4.3	415	<30	<30	<30	<30	<10
			18	8.1	3278	<30	<30	<30	<30	<10
			30	5.4	2654	<30	<30	<30	<30	<10
			60	2.9	1287	<30	<30	<30	<30	<10
			235	1.0	985	<30	<30	<30	<30	ND
**B-17 (26, F, Honduras, mission work**)	None; 1	Naive (not pregnant)	7	4.8	1438	<30	<30	<30	<30	<10
**C-16 (32, F, Honduras, mission work**)	Missionary in Honduras since 2010 and YF-17D vaccine in 2010; 3	Experienced (pregnant)	3	0.6	<30	354	561	455	104	30
			7	3.7	1428	1567	6102	12448	4590	15
			14	9.6	8844	8787	7553	12372	5780	30
			28	9.1	6570	4157	3807	6050	3240	30
			44	7.3	3200	3688	2057	4419	2345	30
			75	4.8	3106	2765	1876	2438	1230	30
			103	3.7	2789	2485	1654	1653	765	30
			**218** ^c^	1.9	2086	354	289	368	326	ND
			332	1.1	1863	318	213	322	286	ND
**D-19 (27, F, Jamaica, family visit**)	Originally from Jamaica; 2	Experienced (pregnant)	11	7.5	9876	6860	5272	8115	8170	60
			26	6.5	8428	5438	4568	6985	6534	30
			33	5.1	7069	4378	3482	5342	5428	30
			52	3.4	3546	3685	2896	3564	4512	20
			80	2.4	1864	3262	2786	2860	2874	<10
			**172** ^c^	2.1	962	658	754	1086	869	ND
			301	0.9	847	527	436	964	548	ND
**E-18 (26, F, multiple Caribbean islands, vacation**)	YF-17D vaccine in 2007; past history of extended travel in tropics; 8	YF-17D experienced (not pregnant)	8	4.2	778	<30	<30	77	88	<10
			18	7.8	4146	55	88	176	156	<10
			35	5.8	1118	50	<30	101	129	<10
			347	1.2	743	<30	<30	<30	<30	ND

Abbreviations: Ab, antibody; DENV, dengue virus; DPO, days post onset of symptoms; FRNT_50_ titer, 50% focus reduction neutralization test titer; Ig, immunoglobulin; MAC-ELISA, IgM antibody-capture enzyme-linked immunosorbent assay; NA, not applicable; ND, not done; PCR, polymerase chain reaction; WNV, west nile virus; ZIKV, Zika virus.

^a^IgM testing was conducted using MAC-ELISA. ELISA values were patient serum optical densities divided by healthy control serum optical densities; <2, negative; 2–3 equivocal; >3 positive

^b^IgG titers were determined as endpoint titers using indirect ELISA

^c^For pregnant patients C-16 and D-19, the DPOs corresponding to delivery are bolded.

**Table 2.** Clinical Signs and Symptoms of 5 Patients with Acute Zika Infection

Patient	Month and Year of Zika Infection	Days Post Onset of Symptoms at Enrollment	Rash (days)	Fever (days)	Conjunctivitis (days)	Joint Pain and/or Swelling (days)	Headache or Eye Pain (days)	Myalgia (days)	Fatigue or Weakness (days)	Nausea or Diarrhea (days)	Cognitive Difficulty (days)	Notes
A-23	August 2016	5^a^	5	9	0	9	8	9	30	1	0	
B-17^b^	May 2016	7	7	6	15	0	18	17	18	17, nausea	0	
C-16	May 2016	3	4	4	0	28	103	2	103	0	0	Patient attributed fatigue to pregnancy
D-19	June 2016	11	4	0	0	0	52	0	0	0	0	Headache began prior to Zika illness
E-18	May 2016	8	8	8	0	6	9	6	94	0	2	Crohn’s disease on intermittent prednisone

^a^On days post onset of symptoms 3 (in hospital emergency room preenrollment) patient A-23 had a low white blood cell count of 1900 cells/μL (normal, 4200–9100 cells/μL ) with reduced absolute lymphocytes (500 cells/μL; normal, 720–3290 cells/μL), elevated reactive lymphocytes (20 cells/μL; normal, 0, an indication of viral infection), and low eosinophils (20 cells/μL; normal, 50–290 cells/μL). He also had elevated creatinine (1.21 mg/dL; normal, 0.7–1.2 mg/dL). These laboratory abnormalities resolved gradually over several days.

^b^As we reported previously [22].

**Figure 1.**
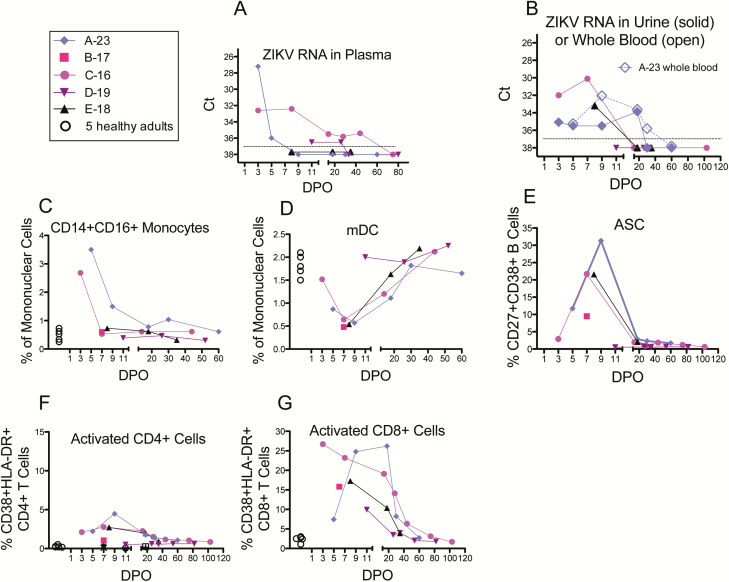
Viral loads and phenotypic assays for innate cells, antibody-secreting cells (ASC), and activated T cells. Colored solid symbols and lines identify the 5 acute Zika patients. Open black circles are control data from 5 healthy adults. *x*-axes for all plots, days post onset of symptoms; *y*-axes, response magnitudes. Monocytes and dendritic cells (DCs) were gated as in Supplementary Figure S1, and their frequencies are expressed as percentages of total mononuclear cells in blood. ASCs and activated T cells were gated as in Supplementary Figure S2. *A,* Zika virus (ZIKV) RNA in plasma. *B,* ZIKV RNA in urine (solid symbols for 4 patients) or whole blood (open diamond; only patient A-23 assessed in this study). *C,* CD14+CD16+ intermediate monocytes. *D,* myeloid DC (mDC). Other monocyte subsets, plasmacytoid DC (pDCs), and natural killer (NK) cells showed no clear dynamic trends (not shown). *E,* ASCs (or plasmablasts). *F,* Activated CD4+ T cells. *G,* Activated CD8+ T cells. Abbreviations: ASC, antibody-secreting cells; Ct, cycle threshold for qRT-PCR reactions; DPO, days post onset of symptoms; HLA-DR, human leukocyte antigen-D related; mDC, myeloid dendritic cells; ZIKV, Zika virus.

*Patient A-23.* A 27-year-old healthy male who was flavivirus naive and had vacationed in Belize (Table 1). Upon return to the United States, his acute illness began (Table 2). He was leukopenic on DPO 3 with gradual resolution. ZIKV RNA was present in plasma on DPOs 3 and 5 (with the detected peak on DPO 3); in semen on DPO 9 (the only day assessed); in urine through DPO 18; and in whole blood through DPO 30 (Figure 1A and B). Fatigue persisted for 1 month.

**Figure 2.**
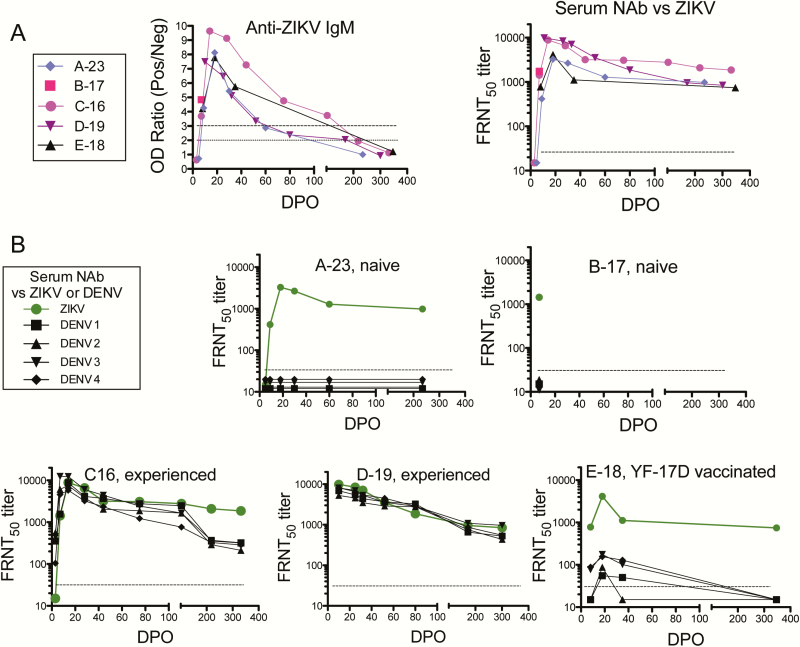
Antibody responses. *A,* Serum anti–Zika virus (ZIKV) immunoglobulin (Ig) M was determined by Zika IgM antibody-capture-enzyme-linked immunosorbent assay (MAC-ELISA) and serum neutralizing antibody (NAb) against ZIKV by focus reduction neutralization test (FRNT). The IgM ELISA values were the ratios of the patients’ serum optical densities to healthy control serum optical densities; <2, negative (lower dashed horizontal line in IgM panel); 2–3, equivocal; >3, positive (upper dashed line). The dashed horizontal line in the NAb panel is the negative cutoff: a titer of <30. The FRNT_50_ titer is the reciprocal of the serum dilution at which a 50% reduction in viral foci was observed. *B,* Serum NAb against ZIKV (green symbol and line) or dengue virus-1–4 (black symbols and lines) in the FRNT. Note: subject E-18 also had a past history of extended travel in the tropics. Abbreviations: DENV, dengue virus; DPO, days post onset of symptoms; FRNT, focus reduction neutralization test; Ig, immunoglobulin; NAb, neutralizing antibody; OD, optical density; ZIKV, Zika virus.

**Figure 3.**
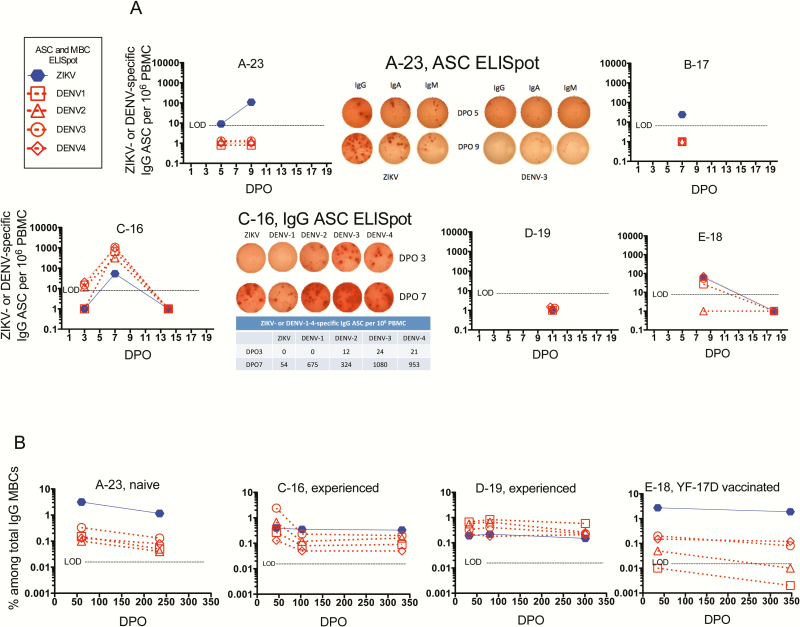
Enzyme-Linked ImmunoSpot (ELISpot) assays for Zika virus (ZIKV)– and dengue virus (DENV)–specific antibody-secreting cells (ASCs) and memory B cells (MBCs). *A,* Antigen-specific ASCs were quantitated using fresh peripheral blood mononuclear cells (PBMCs). For patient A-23, 3 isotypes of ZIKV-specific immunoglobulin (Ig; M, A, and G) were detected in this flavivirus-naive patient (and for patient B-17, also flavivirus-naive). In contrast, for patient C-16 (flavivirus-experienced), only IgG-secreting ASCs were detected; also true of patient E-18 (also flavivirus-experienced). *B,* MBCs against ZIKV or DENV were detected in thawed PBMCs. In these assays absolute levels of ZIKV-specific vs DENV-specific ASCs or MBCs cannot be directly compared since the ZIKV antigen was viral lysate and the DENV antigens were recombinant E proteins. Abbreviations: ASC, antibody-secreting cells; DENV, dengue virus; DPO, days post onset of symptoms; Ig, immunoglobulin; LOD, limit of detection; MBC, memory B cell; PBMC, peripheral blood mononuclear cell; ZIKV, Zika virus.

CD14+CD16+ proinflammatory monocytes were increased and at their detected peak on DPO 5 (when first assessed) and decreased to a presumed baseline by DPO 18 (Figure 1C). Myeloid dendritic cells (mDCs) decreased to a nadir on DPO 9 and increased to a presumed baseline by DPO 30 (Figure 1D). Anti-ZIKV IgM was not detected on DPO 5, was positive on DPOs 9 through 30, decreased to equivocal on DPO 60, and was absent on DPO 235 (Figure 2A, Table 1). Neutralizing antibodies (NAb) against ZIKV was first detected on DPO 9, peaked on DPO 18 (at a titer of 3278) when viral RNA in urine and whole blood were present but decreasing, and by DPO 235 had decreased only 3.3-fold (to 985; Figure 2A, Table 1). NAb against DENV-1–4 and ELISA Ab against WNV were negative in this naive patient (Figure 2B, Table 1). On DPOs 5–18 a striking rise and fall of phenotypic antibody-secreting cells (ASCs) occurred, peaking at 31% of all blood B lymphocytes (Figure 1E). The ASCs’ specificity for ZIKV was demonstrated by ELISpot assay, and there were no anti-DENV-1–4-, WNV-, or yellow fever virus (YFV)-specific ASCs (flavivirus-naive patient; Figure 3A). Interestingly, memory B cells (MBCs) with cross-reactivity against DENV-1–4 developed (Figure 3B).

**Figure 4.**
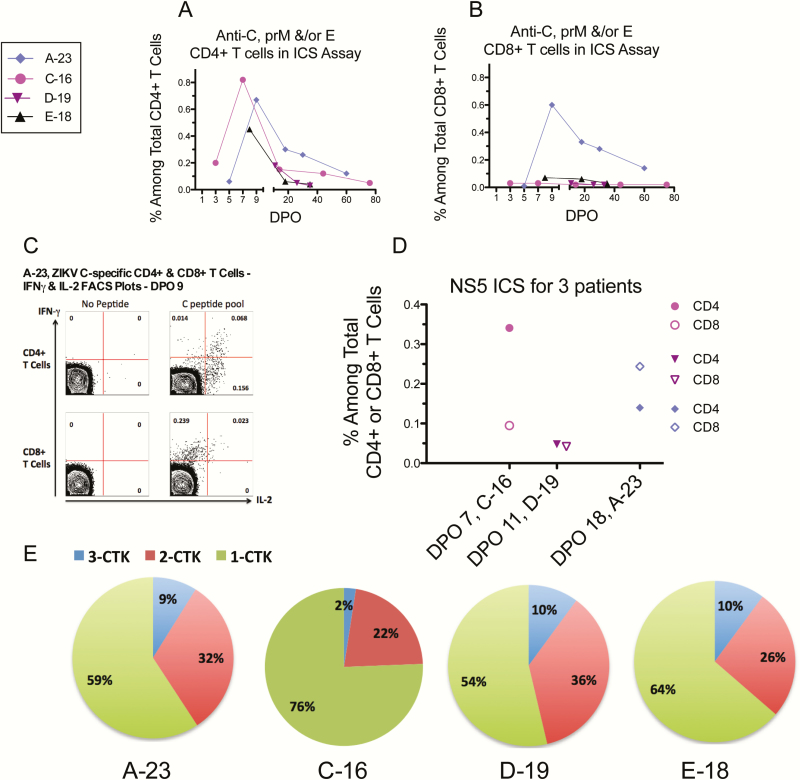
T-cell intracellular cytokine staining (ICS) assays. CD4+ or CD8+ T cells were stimulated for 6 hours with peptide pools spanning Zika virus (ZIKV) C, prM, E, or NS5 proteins. Thawed peripheral blood mononuclear cells from various days post symptom onset were used in these assays. Antiviral cells expressing intracellular cytokines (interferon [IFN]-g, interleukin [IL]-2, and/or tumor necrosis factor-a) were then detected by ICS and flow cytometry. *A* and *B,* ZIKV C-, prM-, and/or E-specific cytokine-producing CD4+ or CD8+ T cells. Summary results are presented here as pooled total cytokine-producing cells analyzed in a Boolean analysis. *C,* FACS plots demonstrating C-specific CD4+ and CD8+ T cells for patient A-23. CD8+ cells produced primarily IFN-g, whereas CD4+ cells produced both IFN-g and IL-2. D. NS5-specific T cells were assessed with available cells for 3 patients. Closed symbols, CD4+ T cells; open symbols, CD8+ T cells. Patent D-19’s result was negative (as defined in Supplementary Table S1 footnote). *E,* Polyfunctionality of the CD4+ T cells. The pie charts display the relative proportions of CD4+ T cells producing 1, 2, and 3 cytokines (CTK) (Boolean analysis); 3 CTK (blue), percentage of cells producing all 3 cytokines; 2 CTK (red), cells producing 2 cytokines; and 1 CTK (green), cells producing a single cytokine. Abbreviations: CTK, cytokine; DPO, days post onset of symptoms; FACS, fluorescence activated cell sorter; ICS, intracellular cytokine staining; IFN, interferon; IL, interleukin; ZIKV, Zika virus.

Activated CD4+ T cells peaked moderately on DPO 9, but CD8+ T cells had a robust and prolonged plateau of activation (up to 26% of total blood CD8+ T cells) before decreasing toward a baseline on DPO 60 (Figure 1F–1G), the first time point when whole blood ZIKV RNA was negative (Figure 1B). Cytokine-producing antiviral CD4+ and CD8+ T cells against ZIKV C, prM, E, and NS5 peptides were demonstrated in ICS assays; first detected on DPO 9 and persisting through DPO 60 (Figure 4, Supplementary Table S1). Also, 41% of the antiviral CD4+ T cells were polyfunctional, that is, expressed ≥2 cytokines (Figure 4E).

*Patient B-17.* Five days after returning from Honduras, a 26-year-old nonpregnant flavivirus-naive woman developed typical Zika symptoms (Tables 1 and 2). ZIKV RNA was present in multiple body fluids (including whole blood through DPO 81, saliva, urine, and vaginal fluid; not shown, previously reported [22]). DENV and chikungunya RNA were not detected in plasma. On DPO 7, proinflammatory monocytes, mDCs, ASCs, and activated T cells were similar to those in patient A-23 (Figure 1C–1G; reported here for the first time). Anti-ZIKV IgM was positive and FRNT_50_ titer against ZIKV was 1438 on DPO 7; there were no NAbs against DENV nor binding Ab against WNV (Table 1, Figure 2A–2B; partially reported previously [22]). ZIKV-specific ASCs were identified using ELISpot, but no DENV-specific ASCs were present in this naive patient (Figure 3A). Cytokine-secreting E-specific CD4+ effector T cells, but not CD8+ T cells, were present (Supplementary Table S1).

*Patient C-16*. A 36-year-old flavivirus-experienced missionary in Honduras was 9 weeks pregnant when she developed Zika (Table 1). Arthralgia persisted for 1 month; headache and fatigue were present through DPO 103, but she attributed fatigue to the pregnancy (Table 2). Plasma ZIKV RNA was positive from DPO 3 through 44 and became negative on DPO 75 (Figure 1A); plasma was negative for DENV and chikungunya RNA. Urine PCRs were positive for ZIKV RNA on DPOs 3 and 7 but not after that (Figure 1B). At delivery (DPO 218) ZIKV PCR of amniotic fluid and cord blood were negative, as was viral culture of the placenta. The newborn appeared healthy and remained so through age 4 months.

Cell phenotyping again revealed a transient increase in proinflammatory monocytes (Figure 1C) and a decrease in mDCs (Figure 1D). Anti-ZIKV IgM was initially negative (DPO 3), high equivocal on DPO 7, then positive through DPO 103, and negative at DPO 218 (delivery; Table 1). On DPO 3, DENV-specific NAb was detected in serum (Figure 2A and 2B, Table 1), but anti-ZIKV NAb was not detected until DPO 7 and then peaked on DPO 14 as plasma ZIKV RNA levels began to decrease. ZIKV- and DENV-specific NAbs were long-lived; for example, on DPO 332 FRNT_50_ titers were 1863 and 322 [DENV-3], respectively. Phenotypic ASCs had a robust DPO 7 peak (22% of all blood B cells; Figure 1E). Like NAb, DENV-specific IgG-producing ASCs were first detected on DPO 3 using ELISpot and later (DPO 7) ZIKV-specific ASCs were detected (Figure 3A). As expected, MBCs against both ZIKV and all DENV serotypes were detected; anti-ZIKV MBCs underwent less decay (Figure 3B).

Moderate CD4+ T-cell activation (Figure 1F) and functional antigen-specific CD4+ T cells against C, prM, E, and NS5 peptides were present (Figure 4A and 4D). Strong CD8+ T-cell activation (27% of total blood CD8+ T cells) was detected early (DPO 3; Figure 1G) and persisted in association with prolonged plasma viral RNA (Figure 1A). However, functional (cytokine-producing) CD8+ T cells were either not detected (against C, prM, and E peptides) or present at only very low levels (NS5) in ICS assays performed repeatedly through DPO 75 (Figure 4B and 4D; Supplementary Table S1). Further phenotyping of the activated but apparently hypofunctional CD8+ T-cell subset identified markers of antigen-driven proliferation, tissue homing, and cytotoxic effector functions, indicating activation via the T-cell receptor (TCR) and not bystander activation (Supplementary Figure S3).

*Patient D-19*. A 27-year-old flavivirus-experienced, healthy, and pregnant US resident traveled to Jamaica to visit a relative. Soon after returning to the United States she developed acute Zika during the sixteenth week of gestation (Tables 1 and 2). Headache persisted for 52 days but had begun prior to her Zika illness. ZIKV RNA was present in plasma on DPOs 11 and 26 and not detected on DPO 33 (Figure 1A). qRT-PCR was negative for DENV and chikungunya RNA in plasma, ZIKV RNA in urine, and ZIKV RNA in amniotic fluid (DPO 33). The newborn appeared healthy and had remained so through 6 months.

Because her first study visit occurred later (DPO 11), monocyte, mDC, and ASC phenotyping did not identify the acute changes observed in other patients (Figure 1C–1E). Her anti-ZIKV IgM was positive through DPO 33; equivocal on DPOs 52, 80, and 172 through delivery; and negative on DPO 301 (Figure 2A, Table 1). High FRNT_50_ titers against both ZIKV and DENV were present on DPO 11 (eg, 9876 against ZIKV) and persisted (Figure 2A–2B, Table 1). Antigen-specific ASC ELISpot on DPO 11 was negative (missed peak; Figure 3A). MBCs against both ZIKV and DENV were present on DPOs 33, 80, and 301 (Figure 3B).

CD4+ T-cell activation was not observed (later initial time-point [DPO 11]; Figure 1F), but CD8+ T cells were activated and remained so through DPO 33 when plasma viral RNA became negative (Figure 1G). Antiviral cytokine-producing CD4+ T cells were detected by ICS; however, in this pregnant patient with prolonged viral RNA in plasma, functional CD8+ T cells against C, prM, E, and NS5 peptides were not detected (Figure 4, Supplementary Table S1).

*Patient E-18*. A 28-year-old nonpregnant woman developed acute Zika upon return to the United States from the Caribbean; she previously received YFV-17D vaccine and she also had a past history of extended travel in the tropics (Tables 1 and 2). On DPO 8 ZIKV RNA was detected in urine but not plasma; then negative in both from DPO 18 onward (Figure 1A-B). Phenotyping of monocyte, mDC, ASC, and activated T-cell populations demonstrated increases and decreases as for the other patients (Figure 1C–1G). IgM was positive on DPOs 8 through 35 and negative on DPO 347 (Figure 2A, Table 1). NAb titers against ZIKV were high and against DENV were low (Figure 2A–B, Table 1). ELISpot assays for ASC and MBC identified ZIKV- and DENV-specific cells (Figure 3). She produced functional effector T cells against ZIKV C, prM, E, and/or NS5 peptides at moderate (CD4+ subset) or low (CD8+ subset) levels (Figure 4, Supplementary Table S1).

## DISCUSSION

Observations that characterize human immune responses to natural Zika infection are of importance for multiple groups that develop and test ZIKV vaccines in clinical trials. With a sample size of 5 it would be foolish to draw definitive or broad conclusions. However, in summarizing the data, a few interesting points for consideration emerge.

ZIKV RNA was detected in plasma from 4/5 patients and in urine from 3/4 patients tested. In 2 patients for whom whole blood was tested, viral RNA persisted longer in blood than in plasma or urine. Prolonged ZIKV RNA detection in patient A-23’s whole blood is consistent with previous reports of flavivirus persistence in whole blood (and viral association with red blood cells) for ZIKV [22, 25] and WNV [26, 27]. Whole blood may therefore provide a wider window for molecular diagnosis of Zika in upcoming vaccine efficacy studies.

DENV and chikungunya RNA were not detected in 3/3 patients tested, excluding coinfections. Despite persistent viral RNA in plasma, 2/2 pregnant women mounted robust phenotypic innate, T-, and B-cell responses; antigen-specific CD4+ T-cell responses; and NAb titers that were similar to those in nonpregnant patients. Both newborns were healthy. Neither pregnant woman had detectable functional CD8+ T-cell responses against ZIKV structural protein peptides (1 had a weak NS5 response). Their prolonged plasma ZIKV RNA was presumably due to shedding from infected placenta [28].

Early (DPO 3–9) transient increases in proinflammatory monocytes and slightly later (DPO 5–18) transient decreases in mDCs occurred. No clear patterns were observed for other monocyte subsets, plasmacytoid DC (pDC) or natural killer cells (NK). Knowledge of these cellular shifts during acute Zika infection may lead to pathways to exploit for vaccine or therapeutic development. Increases in blood CD14+CD16+ intermediate monocytes were also seen in acute dengue patients with high viral loads, and this population of cells was shown in vitro to mediate plasmablast differentiation and antibody production via B-cell activating factor (BAFF)/a proliferation-inducing ligand (APRIL) and interleukin (IL)-10^19^. A decrease in blood mDCs was described for DENV infections where DCs are a major site for viral replication with resulting apoptosis [19, 29]. ZIKV, like many flaviviruses, infects human DCs [30, 31]. ZIKV antagonizes DC interferon responses in vitro [31].

Strong activation of CD8+ T cells (15% to >25% of blood CD8+ T cells) developed early and persisted in parallel with viral RNA. Moderate activation of CD4+ T cells (2%–5%) occurred. Lower, similar, and higher levels of T-cell activation were reported for YFV-17D, DENV, and Ebola acute infections, respectively [20, 32, 33].

Four patients studied before DPO 10 had robust phenotypic ASC responses (up to 31% of blood B cells). In flavivirus-experienced patients, ASCs against both ZIKV and DENV were present; in flavivirus-naive patients, only ZIKV-specific ASCs developed. This same pattern was seen for NAb. In experienced patients, ASC and NAb to DENV appeared earlier than those to ZIKV, emphasizing that NAb assays alone cannot be used for ZIKV diagnosis in flavivirus-experienced patients. Titers of ZIKV- and/or DENV-specific neutralizing Ab had impressive longevity (eg, DPO 332, patient C-16, titer 1863), as has been reported for other flaviviruses [34, 35]. ZIKV-specific MBCs were higher in flavivirus-naive patients—perhaps due to freedom from original antigenic sin. The development of MBC cross-reactive to DENV following Zika in a DENV-naive patient (eg, patient A-23) raises a theoretical possibility of antibody-dependent enhancement in a subsequent (first) DENV infection for such patients or for ZIKV vaccine recipients. Larger studies are needed to further address these observations.

In addition to 2 case reports we published recently [9, 36], we were unable to identify publications on ICS assays during human Zika infections. C-, prM-, E-, and NS5-specific cytokine-expressing CD4+ T cells developed in all patients tested, and 22%–46% of the responding cells were polyfunctional (Figure 4E). The generally absent or low detection of functional CD8+ effector T cells against peptides spanning ZIKV C, prM, E, and NS5 in this study is of interest. Further studies in larger samples and with peptides that span all 10 ZIKV proteins are needed. The high frequencies of activated CD8+ T cells compared to the absent or low magnitude of functional ZIKV-specific CD8+ effector T cells (eg, activated-to-functional ratio of 41:1 in patient A-23) was striking. In persistent viral infections (eg, human immunodeficiency virus, hepatitis C virus, lymphocytic choriomeningitis virus) and cancer, chronic antigen exposure causes CD8+ T-cell exhaustion (dysfunction) [37, 38]. In our study, the highly activated CD8+ T cells carried markers of exposure to antigen via TCR, which argued against bystander activation, yet few CD8+ T cells specific for the tested peptides were found.

In one study of dengue patients, the vast majority of proliferating, highly differentiated effector CD8+ T cells had acquired TCR refractoriness with deficient cytokine production. Transcriptomics revealed downregulation of TCR signaling molecules [32], leading the authors to infer that acute dengue’s massive CD8+ T-cell activation leads to downregulation of TCR signaling, producing a stunned phenotype previously described for other viruses [37, 38]. Also, with other flaviviruses, the structural proteins are predominantly targeted by CD4+ more than CD8+ T cells [39, 40]. The limited ZIKV-specific CD8+ T-cell response in pregnancy observed here deserves further study.

Finally, the public health significance of ZIKV RNA persistence in body fluids needs to be confirmed with culture or molecular assays that quantify replication-competent virus (a gap in the ZIKV clinical literature). Such efforts are underway in our laboratory. The clinical, viral, and immunologic findings in this small series of Zika patients may be of particular interest to clinicians and scientists planning vaccine studies.

## Supplementary Data

Supplementary materials are available at *Clinical Infectious Diseases* online. Consisting of data provided by the authors to benefit the reader, the posted materials are not copyedited and are the sole responsibility of the authors, so questions or comments should be addressed to the corresponding author.

## Supplementary Material

Suppl_Mat_Lai_et_al_CID_86907_uploaded_072417

## Notes

***Acknowledgements.*** The authors thank the study volunteers for their generous participation. For helpful scientific discussions we thank Toby Merlin, Walla Dempsey, Robert Johnson, Jens Wrammert, Mehul Suthar, and Rafi Ahmed. Thanks to Jesse Waggoner for performing semen PCR. We appreciate the contributions of the Georgia Department of Public Health; and the clinical contributions of Lara Harik, John P Horton, and Martina Badell from the Emory University Departments of Pathology, Obstetrics and Gynecology, and Maternal Fetal Medicine.

The Emory Zika Patient Study Team includes the byline coauthors and the following individuals: Briyana Domjahn, MPH, Dongli Wang, BS, Mary Bower, RN, Rijalda Deovic, BS, Sree Aramgam, BS, Sara Jo Johnson, BS, Dean Kleinhenz, BS, JoAnn Sadowski, BS, Talib Sirajud-Deen, BA (from Hope Clinic of the Emory Vaccine Center, Division of Infectious Diseases, Department of Medicine, School of Medicine, Emory University, Decatur, Georgia), and Jesse Waggoner, MD (from Hope Clinic of the Emory Vaccine Center, Division of Infectious Diseases, Department of Medicine, School of Medicine, Emory University, Decatur, Georgia and Emory TravelWell Center, Division of Infectious Diseases, Department of Medicine, School of Medicine, Emory University, Atlanta, Georgia).

***Financial support.*** This work was supported by the Emory University School of Medicine; Emory Vaccine Center; Georgia Research Alliance; and the National Institutes of Health (NIH), National Institute of Allergy and Infectious Diseases (NIAID), Division of Microbiology and Infectious Diseases (DMID; contract HHSN272201300018I) and by a supplemental award to NIH/NIAID/DMID (grant AI115651).

***Potential conflicts of interest.*** H. W. has received grant support from Takeda Vaccines, outside the submitted work. All other authors: No reported conflicts of interest. All authors have submitted the ICMJE Form for Disclosure of Potential Conflicts of Interest. Conflicts that the editors consider relevant to the content of the manuscript have been disclosed.
