# Supplementary material for: Innate, T-, and B-Cell Responses in Acute Human Zika Patients
Source: Clin Infect Dis. 2017 Aug 17;66(1):1–10. doi: 10.1093/cid/cix732 (PMC5850027; doi:10.1093/cid/cix732)
Supplement: Suppl_Mat_Lai_et_al_CID_86907_uploaded_072417 [file cix732_suppl_suppl_mat_lai_et_al_cid_86907_uploaded_072417.docx]

**Supplemental Materials**

**Innate, T and B Cell Responses in Acute Human Zika Patients**

**Lai et al.**

**Additional Details on Materials and Methods for the Assays**

**Innate, T, and B Cell Phenotyping Assays** Monoclonal antibodies were used for staining of fresh whole blood for innate cell phenotyping: from BD, CD3 (UCHT1, #557943), CD19 (HIB19, #557921), CD14 (M5E2, #565283), HLA-DR (G46-6, #560651), CD11c (O33-782, #561355) and CD123 (7G3,554529); from eBiosciences, CD56 (MEM188,17-0569) and CD16 (CB16,47-0168); and from Biolegend: CD20 (2H7, #302322). For T and B cell activation: from BD, CD3 (SP34-2, #562877), CD4 (L200, #560836), CD8 (SK1, #565310), CD19 (HIB19, #555415), CD38 (HIT2, #555460), and HLA-DR (G46-4, #555811); from eBiosciences: CD20 (2H7, #47-0209042); and from Biolegend, CD27 (O323, #302838). Data were collected on an LSRII (BD Biosciences) and analyzed using FlowJo software (Tree Star).

**Virus Neutralization Assay and ELISAs** Serum neutralizing antibodies against ZIKV or DENV-1-4 were measured by focus reduction neutralization test (FRNT) as described^1^ with selected modifications. Serially diluted, heat inactivated sera were incubated with a previously titrated amount (60–100 focus forming units) of ZIKV or DENV-1-4 for 1h at 37 °C. Vero cell monolayers in 96-well plates were infected with the mixture for 1h at 37 °C. An overlay containing 2% (wt/vol) methylcellulose (Sigma; M0512-2506) was added to the cells. After a 3 day incubation at 37 °C, the cells were washed and fixed with a 1:1 mixture of acetone and methanol. Foci were stained with a pan-flavivirus mouse monoclonal D1-4G2-4–15 (MAB10216 EMD Millipore) for 2 h followed by HRP-linked anti-mouse IgG (Cell Signaling; 7076S) for 1h and developed using TrueBlue peroxidase substrate (KPL; 50–78-02). Foci were imaged using a CTL-Immunospot S6 Micro Analyzer and counted.

Serum anti-ZIKV IgM antibodies were detected by the Zika IgM Antibody Capture Enzyme-Linked Immunosorbent Assay (Zika MAC-ELISA) as described^2,3^.

West Nile Virus (WNV) IgG antibodies were detected using an endpoint ELISA adapted from a previously described method^1,4^. Briefly, Nunc Maxisorb plates were coated with 1 μg/ml WNV Recombinant E Protein (Mybiosource, MBS5304480) in 1X PBS and incubated at 37 °C for 30 min, then overnight at 4 °C. Plates were washed in TBST, blocked in 5% Dry Milk/TBST, and incubated with diluted sera. Horseradish peroxidase conjugated goat anti-human IgG was used to detect WNV-specific IgG antibodies and developed with KPL TMB substrate. Endpoint titer was determined as the last dilution >2x the optical density of the healthy control serum.

**Antigen-specific B Cell ELISpot Assays**

**Antibody-secreting cells (ASCs; plasmablasts)**  Direct ELISpot quantified antigen-specific IgM, IgA or IgG ASCs present in fresh PBMC. Ninety-six well filter plates (Millipore, #MSHAN4B50) were coated with whole cell lysate from Vero cells infected (or not) with Zika virus (1:200 lysate dilution) or 100 ng per well of DENV E proteins (CTK Diagnostics). The plates were left to adsorb overnight at 4°C, washed four times in PBS, and incubated in RPMI medium with 10% (vol/vol) FBS for 30 min at 37°C. RPMI was removed, and fresh PBMCs suspended in RPMI with 10% FBS were placed in each well with threefold dilutions. Plates were incubated at 37°C overnight, then were washed four times in PBS with 0.05% Tween 20 (PBST) and incubated for 2h at room temperature with biotinylated anti-human IgG (Jackson Immunoresearch Laboratory, #709065098) at 1 μg/mL diluted in PBST with 1% FBS. Plates were washed four times in PBST and incubated for 1h at room temperature with streptavidin-HRP (Vector Laboratories #A 2004) diluted 1:1,000 in PBST with 1% FBS. Plates were washed three times each in PBST, then with PBS, followed with incubation with 3-Amino-9-ethylcarbazole (AEC) substrate kit (EMD Millipore Corporation, Substrate #152226, Buffer #152224) for 10 min until spot development. Plates were washed with water and allowed to dry; images were obtained using a CTL ELISpot plate reader.

**Memory B cells (MBCs)** were detected as described^5^ by incubating thawed PBMCs at 5×10^6^ cells per mL in R-10 supplemented with IL-2 and R488 (CTL-hBPOLYS-200, CTL) plus antigens for 6 days. Total and virus-specific IgG-secreting MBCs were then quantified by ELISpot assay as described above.

**Intracellular Cytokine Staining** Thawed PBMCs were rested overnight and then incubated for 6h at 37°C with viral peptide pools at a final concentration of 2 μg/mL for each peptide in the presence of anti-CD28 and -CD49d (diluted 1:100; BD, # 555725 and #555501) and GolgiPlug (1:1,000 dilution; BD, #555029). Negative-control samples were left unstimulated and positive-control samples were treated with Staphylococcal enterotoxin B (Sigma, #S4881) at a final concentration of 1 μg/mL or Cell Stimulation Cocktail containing phorbol 12-myristate 13-acetate (PMA) and ionomycin (#00-4970-03, eBiosciences). PBMCs were then stained with Zombie cell viability dye (L423102, Biolegend). After fixation/permeabilization with Cytofix/Cytoperm, cells were stained with antibodies against: CD3, CD4, and CD8 as above; IL-2 (MQ1-17H12, #554567) and TNF-α (MAB11, #550679) from BD; and IFN-gamma (4S.B3, #47-731942) from eBioSciences. After the cells for phenotyping and (ICS) cytokine staining experiments were washed, data were collected on an LSRII (BD Biosciences) and analyzed using FlowJo software (Tree Star) as described^6^. Gating strategies are provided in Figures S1 and S2.

**Other Antigens** For B cell ELISpot assays, DENV-1-4 recombinant envelope (E) proteins were purchased from CTK Diagnostics (A2301, DENV-1 VN/BID-V949/2007; A2302, DENV-2 GWL39 IND-01; A2303, DENV-3 US/BID-V1090/1998; and A2304, DENV-4 341750). WNV E protein was purchased from My Biosource (MBS 5304480). YF-VAX (YF-17D) Vaccine (Sanofi) was used as YFV antigen. A pan-flavivirus mouse monoclonal D1-4G2-4–15 (MAB10216) was purchased from EMD Millipore.

**qRT-PCR** for Zika diagnosis and ZIKV persistence employed the following primer sets (GenBank: EU545988.1).

Zika1087F: CCGCTGCCCAACACAAG

Zika1107FAM: AGCCTACCTTGACAAGC AGTCAGACACTCAA

Zika1162R: ATGTCTGCAAAAGAACGTTAGTGG

Zika4481F: CTGTGGCATGAACCCAATAG

Zika4507RFAM: CCACGCTCCAGCTGCAAAGG

Zika4552R: ATCCCATAGAGCACCACTCC

**Supplemental Figures S1-S3 and Table S1**

**Figure S1.** **Gating strategy for innate cells.**  Representative dot plots from fresh whole blood showing innate cells subsets in fresh whole blood of Zika patients. Single cells were selected on the basis of forward scatter area and height characteristics (FSC-A and FSC-H). **A. Monocytes** were identified within the SSC-A-hi FSC-A-hi cells as the CD3-CD19-CD20-CD56- HLA-DR+ population and gated for the CD14+CD16-, CD14+CD16+, and CD14dimCD16++ subsets. **B. NK cells** were identified within the CD3-CD19-CD20-CD14- as a CD56+CD16+ double positive population. **C. DCs.** Myeloid DCs (mDC) were gated within the Lineage (Lin.: CD3, CD14, CD16, CD19, CD20, CD56)-negative, HLA-DR+ CD11c+ cells; plasmacytoid DCs (pDC) were identified as Lineage-negative HLA-DR+ CD123+ cells.

**CD3CD19CD20**

**CD14**

**CD16**

**CD14+CD16-**

**CD14+CD16+**

**CD14dimCD16+**

**(A). monocytes**

**(B). NK cells**

**(C).** **DC**

**SSC**

**FSC**

**After**

**single**

**cell**

**selection**

**CD3CD19CD20**

**CD14**

**HLA-DR**

**CD11c**

**CD123**

**Lin- HLA-DR+**

**pDC**

**mDC**

**CD56**

**CD16**

**CD56**

**HLA-DR**

**Figure S2. Gating strategy for T and B cells.** Representative dot plots from fresh whole blood showing T and B cell subsets. Single cells were selected on the basis of forward scatter area and height characteristics (FSC-A and FSC-H). **A. Activated (HLA-DR^+^CD38^+^) T cells.** CD3^+^CD4^+^CD8^−^ cells and CD3^+^CD4^−^CD8^+^ cells were defined as CD4 and CD8 T cells separately. B. **ASCs (plasmablasts).** Total B cells were defined as both CD19 and CD20 expressing cells following CD3 (T lymphocyte) exclusion. ASCs were identified as CD27hi/CD38hi cells among total B cells.

**CD4**

**FSC-A**

**SSC-A**

**CD8**

**CD38**

**HLA-DR**

**Lymphocytes**

**CD8 T cells**

**CD4 T**

**cells**

**HLA-DR^+^CD38^+^**

**CD8 Cells**

**CD3**

**HLA-DR^+^CD38^+^**

**CD4 Cells**

**T cells**

**Non T cells**

**Total B cells**

**CD19**

**CD20**

**CD27**

**CD27^hi^**

**CD38^hi^**

**B cells**

**CD38**

**A**

**B**

**After**

**single**

**cell**

**selection**

**Figure S3. Further phenotyping of the activated CD8+ T cell subset for patient C-16.** DPO 7 is shown on the top row and DPO 28 on the bottom row. Black curves indicate the activated subset of CD8+ T cells (CD38+HLA-DR+); grey curves indicate the subset of unactivated CD8+ T cells (CD38-HLA-DR-). Within each panel, the two numbers shown are the percentages of activated CD8+ T cells with lower or higher fluorescence intensity relative to the small vertical indicator on the horizontal line. During the acute illness phase (DPO 7), compared to the unactivated cells, the activated CD8+ T cells had up-regulated expression of: Ki-67 (a marker of active cell proliferation); CX3CR1 (the fracktalkine receptor for homing to sites of inflammation); PD1 (the strong negative costimulatory molecule); and granzyme B (cytotoxic effector cell molecule). Also on DPO 7 the activated CD8+ T cell subset had down-regulated: CD45RA, CD127 (interleukin 7 receptor IL-7R); and the anti-apoptotic molecule Bcl-2 – all consistent with an effector cell population activated by antigen via the T cell receptor. During the early convalescent phase (DPO 28, bottom row) as the activated proportion of CD8+ T cells decreases these phenotypic markers begin to reverse; e.g., lower Ki-67, CX3CR1, and granzyme B expression; and higher CD45RA and BCl-2 expression.

Additionally, PBMCs from DPO 7 produced cytokines upon TCR-independent stimulation with SEB or PMA-ionomycin; and after polyclonal TCR-dependent stimulation with anti-CD3 plus anti-CD28 (not shown).

**Ki-67**

**CD45RA**

**CD127**

**Bcl-2**

**CX3CR1**

**PD-1**

**Granzyme-B**

10.7%

17.5%

CD38

HLA-DR

DPO 28

DPO 7

**Table S1. ICS Assays with ZIKV Peptide Pools Representing E, C, PrM and NS5: Expression of IFN-ϒ, IL-2 and TNF-α Cytokines at Peak Response Time-points**

|  | | | **% Cytokine + among all CD4+ T cells** | | | **% Cytokine + among all CD8+ T cells** | | |
| --- | --- | --- | --- | --- | --- | --- | --- | --- |
| **Patient** | **DPO** | **Peptide pools** | **IFN-γ** | **IL-2** | **TNF-α** | **IFN-γ** | **IL-2** | **TNF-α** |
| **A-23** |  | E | 0.253 | 0.189 | 0.070 | 0.265 | 0.077 | 0.000 |
|  | 9 | C | 0.160 | 0.083 | 0.027 | 0.199 | 0.040 | 0.045 |
|  |  | prM | 0.042 | 0.035 | <0.02 | 0.013 | 0.050 | <0.020 |
|  |  | E | 0.091 | 0.126 | 0.063 | 0.165 | 0.050 | <0.020 |
|  | 18 | C | 0.037 | 0.056 | <0.020 | 0.064 | <0.010 | 0.047 |
|  |  | prM | <0.010 | <0.030 | <0.020 | 0.005 | <0.010 | 0.000 |
|  |  | NS5 | 0.069 | 0.050 | 0.054 | 0.174 | 0.045 | 0.085 |
| **B-17** | 7 | E | 0.039 | <0.030 | 0.120 | <0.030 | <0.010 | <0.020 |
| **C-16** | 7 | E | 0.059 | 0.098 | 0.068 | <0.030 | <0.010 | <0.020 |
|  |  | C | 0.050 | 0.032 | 0.224 | <0.030 | <0.010 | <0.020 |
|  |  | prM | <0.010 | 0.046 | 0.354 | <0.030 | <0.010 | <0.020 |
|  |  | NS5 | 0.044 | 0.065 | 0.292 | 0.034 | 0.026 | 0.029 |
|  | 14 | E | 0.074 | <0.030 | 0.117 | <0.030 | <0.010 | <0.020 |
|  |  | C | 0.077 | 0.038 | 0.062 | <0.030 | <0.010 | <0.020 |
|  |  | prM | 0.023 | <0.030 | 0.024 | <0.030 | <0.010 | <0.020 |
| **D-19** | 11 | E | 0.024 | 0.050 | 0.024 | <0.030 | <0.010 | <0.020 |
|  |  | C | 0.053 | 0.091 | <0.020 | <0.030 | <0.010 | <0.020 |
|  |  | prM | <0.010 | <0.030 | <0.020 | <0.030 | <0.010 | <0.020 |
|  |  | NS5 | 0.030 | 0.034 | 0.022 | <0.030 | <0.010 | <0.020 |
| **E-18** | 8 | E | 0.214 | 0.278 | <0.020 | 0.034 | 0.022 | 0.025 |
|  |  | C | 0.038 | 0.060 | 0.007 | <0.030 | <0.010 | <0.020 |
|  |  | prM | 0.137 | 0.145 | 0.036 | <0.030 | <0.010 | <0.020 |

Due to PBMC availability not all peptide pools could be tested at all time-points. DPO, days post-onset of symptoms. Values in shaded boxes are negative; i.e., below the negative cut-offs for IFN-ϒ, IL-2 or TNF-α detection (negative cut-off for each cytokine was established as the mean value + 2 standard deviations for the no peptide control stimulations for all five patients).

**References for Supplemental Materials**

1. Priyamvada L, Quicke KM, Hudson WH, et al. Human antibody responses after dengue virus infection are highly cross-reactive to Zika virus. Proc Natl Acad Sci U S A 2016;113:7852-7.

2. Rabe IB, Staples JE, Villanueva J, et al. Interim Guidance for Interpretation of Zika Virus Antibody Test Results. MMWR Morbidity and mortality weekly report 2016;65:543-6.

3. Zika MAC-ELISA. Instructions for Use. CDC, 2016. (Accessed May 13, 2017, at <http://www.cdc.gov/zika/pdfs/zika-mac-elisa-instructions-for-use.pdf>.)

4. Thornburg NJ, Ray CA, Collier ML, Liao HX, Pickup DJ, Johnston RE. Vaccination with Venezuelan equine encephalitis replicons encoding cowpox virus structural proteins protects mice from intranasal cowpox virus challenge. Virology 2007;362:441-52.

5. Crotty S, Felgner P, Davies H, Glidewell J, Villarreal L, Ahmed R. Cutting edge: long-term B cell memory in humans after smallpox vaccination. J Immunol 2003;171:4969-73.

6. Lai L, Davey R, Beck A, et al. Emergency Postexposure Vaccination With Vesicular Stomatitis Virus-Vectored Ebola Vaccine After Needlestick. JAMA 2015;313(12):1249-55.
